# A rare case of fetal spondylocostal dysostosis - prenatal diagnosis and perinatal care in a patient with multiple large leiomyomas

**Published:** 2013-03-25

**Authors:** M Cirstoiu, O Munteanu, O Bodean, C Cirstoiu

**Affiliations:** * Obstetrics and Gynecology Department, Bucharest Emergency University Hospital; ** Orthopedics and Trauma Department, Bucharest Emergency University Hospital

**Keywords:** spondylocostal dysostosis, Jarcho-Levin syndrome, hemivertebrae, ultrasound

## Abstract

The spondylocostal dysostosis (SCD) is one of the two major clinico-radiological subtypes of the Jarcho-Levin syndrome (JLS). The JLS is a rare heterogeneous entity characterized by facial dysmorphism, short-neck, short-trunk, normal sizes limbs, with multiple vertebral anomalies at all levels of the vertebral column and costal defects. The JLS has been classified into 2 major clinical phenotypes, based on the extent and distribution of skeletal anomalies, the pattern of inheritance and the prognosis. We report the case of a non-consanguineous 35-year-old female patient, with a history of multiple large leiomyomas gravida 1, para 1. A three-dimensional ultrasound at 18 weeks of gestation revealed: thoracic and lumbar hemivertebrae with abnormal alignment of the vertebral bodies and kypho-scoliosis, also the absence of two right ribs and abnormal shaped ribs. The biometric measurement was appropriate for gestational age and no other malformations were found. Although there was no previous history, based on the three-dimensional ultrasound findings a mild subtype of JLS was suspected. At term, the patient gave birth, by Cesarean section, to a male fetus, with a weight of 2700g, a length of 50cm and a calculated Apgar score of 9. The postpartum examination of the fetus confirmed the diagnose of SCD. The evolution of the newborn was good - he had no respiratory difficulty; he will benefit from an experimental surgery involving expandable titanium ribs. Our case illustrates the importance of an accurate ultrasound examination, which can be hindered by multiple large leyomiomas, in order to diagnose and to differentiate the two subtypes of JLS. The SCD can have a favorable evolution with the appropriate perinatal and postpartum care.

## Introduction

The spondylocostal dysostosis (SCD) is one of the two major clinico-radiological subtypes of the Jarcho-Levin syndrome (JLS). The JLS is a rare heterogeneous entity characterized by facial dysmorphism, short-neck, short-trunk, normal sizes limbs, with multiple vertebral anomalies at all levels of the vertebral column and costal defects. This syndrome was first described by Jarcho and Levin in 1938, but in 1978 Solomon et al classified it into 2 major clinical phenotypes, based on the extent and distribution of skeletal anomalies, the pattern of inheritance and the prognosis [**1,3**]. Spondylothoracic dysostosis (STD) is an autosomal recessive disorder, in which the ribs themselves have no defects and all fuse symmetrically at the costovertebral joints, but with segmentation and formation defects of the vertebrae throughout the spine giving a classical “crab-like" appearance of the thorax; it has a high incidence of neural tube defects and a higher mortality rate [**3**]. The second subtype of JLS is SCD, which can be inherited in both autosomal dominant and recessive forms, is characterized by intrinsic rib anomalies and it is less likely to have associated neural tube defects [**3**].

## Case presentation

We report the case of a non-consanguineous 35-year-old female patient, with no family history of any congenital anomalies or hereditary diseases and no history of radiation or teratogenic exposure during early pregnancy. The medical history revealed that she was diagnosed with multiple large leiomyomas from the age of 30. With 4 years before pregnancy she underwent uterine artery embolization in order to relieve symptoms caused by the numerous, large, uterine fibroids. With 3 years before pregnancy, a myomectomy was performed and a very large (11/9/6cm) intramural fibroid of the uterus was removed. The 36-year-old male partner, non-consanguineous, had no family history of any congenital anomalies or hereditary disease, no personal medical antecedents, or preconception history of radiation or teratogenic exposure. 

**Fig. 1:**
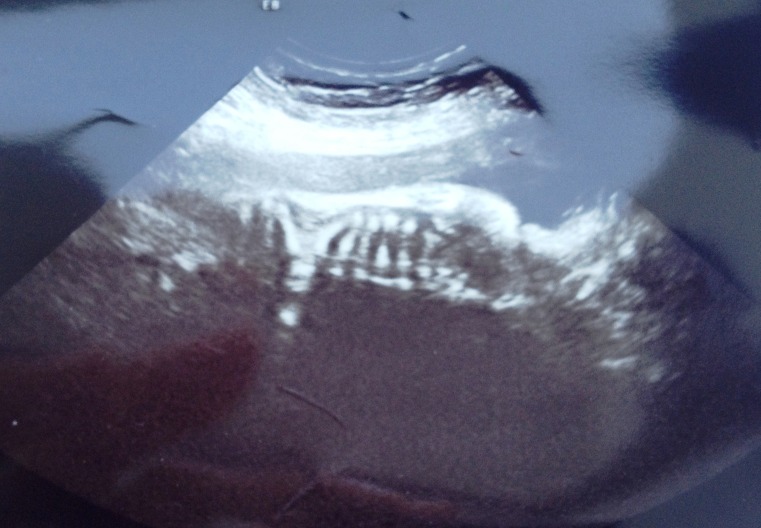
At 18 weeks of gestation – ultrasound aspect in a sagittal section - note thoracic and lumbar hemivertebrae with abnormal alignment of the vertebral bodies and kypho-scoliosis

 The patient, gravida 1, para 1, underwent screening tests and routine ultrasound at 6 and 12 weeks of gestation but no anomalies have been detected. Prior maternal serum screening for alpha-fetoprotein, hCG and UE3 was unremarkable. However, a three-dimensional ultrasound at 18 weeks of gestation revealed thoracic and lumbar hemivertebrae with abnormal alignment of the vertebral bodies and kypho-scoliosis, also the absence of two right ribs and abnormal shaped ribs (**Fig. 1**).

 Because of the short trunk and the limb lengths within normal ranges for gestational age, these appeared relatively long. The biometric measurement was appropriate for gestational age and no other malformations were found. An amniocentesis was performed, but chromosome analysis of amniotic-fluid cells showed a normal karyotype. Although there was no previous history, based on the three-dimensional ultrasound findings a mild subtype of JLS was suspected. Both parents were informed about the detections and the suspicion of SCD; they were informed of the pattern of inheritance and the associated prognosis and they have elected to prosecute the pregnancy. 

 The patient underwent screening tests and routine ultrasounds at 24, 28, 32, 36 and 38 weeks of gestation, but no abnormalities were found. The three-dimensional ultrasound at 24, 28 and 32 weeks of gestation did not reveal different anomalies than the ones already described.

**Fig. 2:**
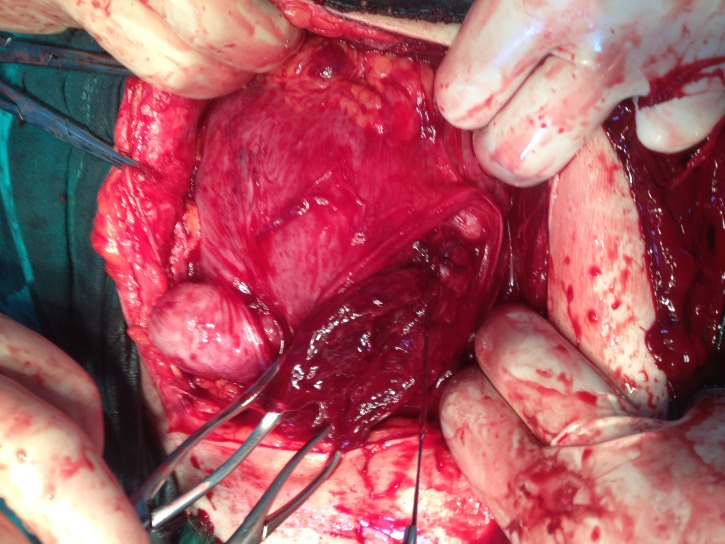
Intraoperative aspect of the uterus – note numerous, large leiomyomas located subserosal and intramural

 At term, the patient gave birth, by Cesarean section, to a male fetus, with a weight of 2700g, a length of 50cm and a calculated Apgar score of 9. During surgery, we have confirmed the presence of the ultrasound diagnosed, numerous, large leiomyomas located subserosal and intramural (**Fig. 2**).

**Fig. 3:**
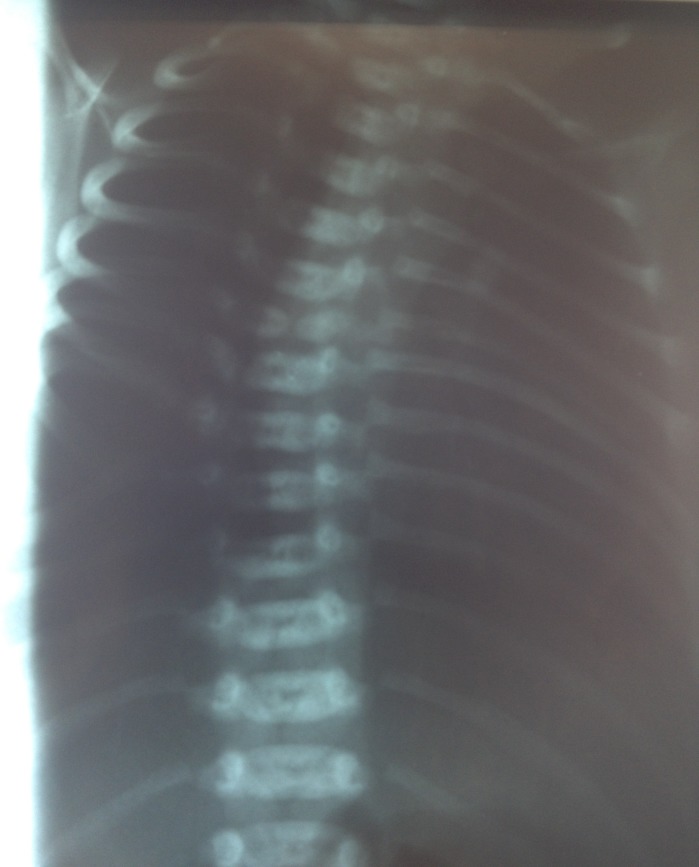
Infantogram showing: thoracic and lumbar hemivertebrae with misalignment of the vertebral bodies, abnormal shaped ribs, the absence of the anterior arches of the 9th and 10th ribs. with moderate scoliosis and kyphosis

The evolution of the newborn was good; he was admitted in the Neonatal Intensive Care Unit for observation, but he had no respiratory difficulty. The postpartum examination showed a mild facial dysmorphism with ears low set, short trunk with protuberant abdomen and relatively long appearing limbs. Cranial ultrasound and abdominal sonography were normal. Radiological findings confirmed thoracic and lumbar hemivertebrae with misalignment of the vertebral bodies, abnormal shaped ribs, and the absence of the anterior arches of the 9th and 10th ribs. with moderate scoliosis and kyphosis (**Fig. 3**). Blood biochemistry showed a normal liver and renal function and also normal blood gases. 

 All these elements were consistent with a mild subtype of JLS – SCD. The newborn will benefit from an experimental surgery involving expandable titanium ribs. At a 6-month follow up visit, the offspring had apparent normal intelligence and no neurological abnormalities. 

 Genetic counseling was offered to the couple for both autosomal dominant and recessive forms of inheritance. These findings were discussed with the parents and plan made to assess any subsequent pregnancy with early screening tests and ultrasound in order to determine if, and what subtype, of JLS is suspected in order to determine the prognosis.

## Discussions

JLS is a rare heterogeneous congenital disease. Cases may be sporadic of familial, with both autosomal dominant and autosomal recessive modes of inheritance reported [**4**]. This disorder has been noted in both consanguineous and non-consanguineous families [**3**]. The STD is found more frequently in people of Puerto Rican descent [**3,4**]. As in our case reported, the SCD is found most often in Caucasians [**4**]. Males and females are affected with equal frequency [**4,5**]. 

 JLS is an eponym that represents a variety of short-neck, short-trunk skeletal dysostosis with vertebral and rib anomalies. The vertebral anomalies described are – hemivertebrae, absent vertebrae, fused vertebrae, block of vertebrae, sickle shaped vertebrae due to segmentation and formation defects [**2,3,6,7**]. The rib malformations determine a classical “crab-like" or “fan-like" appearance of the thorax due to absent ribs, crowded ribs, posterior fusion, irregular shaped, or bifurcation of ribs [**3,8**]. The anomalies associated with JLS include: hernias, neural tube defects, minor facial dysmorphism, anomalies of the digit and the lower limbs, urogenital and anal malformations and complex congenital heart disease [**3,8-11**]. Schulman et al. also described airway abnormalities in two patients with the JLS and consider that these are also an important factor contributing to the respiratory failure of the affected newborns [**12**].

 The JLS was classified by Solomon et al into 2 major clinical phenotypes, based on the extent and distribution of skeletal anomalies, the pattern of inheritance and the prognosis [**2,3**]. 

 SCD can be inherited in both autosomal dominant and recessive forms. The patients are known to have mutations on the DLL3, MESP2 and LFNG genes [**3,13-16**]. It is characterized by intrinsic rib anomalies in combination with multiple segmentation defects of the vertebrae [**3**]. It is clinically defined by a minor facial dysmorphism, a short trunk in proportion to height, short neck; and non-progressive mild scoliosis in most affected individuals [**15**]. This phenotype has the following radiological features: abnormal segmentation of vertebrae, a moderate degree of scoliosis and kyphosis, absent ribs, posterior fusion, irregular shaped, or bifurcation of ribs, with misalignment; but with a preserved general symmetry of the thorax [**3,8,15**]. The SCD is less likely to have associated neural tube defects and other severe anomalies but the males appear to have an increased risk of inguinal hernia [**3,15**].

 In most cases, the evolution is favorable with the adequate intensive care support needed, because in neonates the respiratory function can be compromised due to reduced size of the thorax. [**3,8,15**]. By the age of two years, lung growth may improve sufficiently to support relatively normal growth and development; however, even then life-threatening complications can occur, especially pulmonary hypertension in children with severely restricted lung capacity from birth [**15**]. 

 The second subtype of JLS is STD, an autosomal recessive disorder, associated with mutations in the MESP2 gene [**3,16,17**]. In STD, the ribs themselves have no defects and all fuse symmetrically at the costovertebral joints, but with segmentation and formation defects of the vertebrae throughout the spine giving a classical “crab-like" appearance of the thorax [**3**]. It is defined clinically by: facial dysmorphism, a short-neck, short-trunk, protuberant abdomen, severe scoliosis, inguinal and umbilical hernias and severe anomalies pointed above [**3,8,17**]. The distinctive radiographic findings are: abnormal segmentation of all vertebral segments with characteristic “sickle shaped vertebrae" and severe shortening of the spine but with no intrinsic rib anomalies; the ribs usually appear straight and neatly aligned without points of fusion along their length, determining a characteristically “crab-like" or “fan-like" appearance of the thorax [**3,8,18**]. STD has a high incidence of neural tube defects [**3**]. STD has a higher mortality rate than SCD due to the severe restrictive lung disease and its attendant pulmonary hypertension in neonates, but also because of the stertorous associated anomalies [**3**].

 Most authors consider the severe form of JLS a uniformly lethal condition. The prenatal diagnosis of both STD and SCD phenotypes is possible using fetal ultrasound and in particular - three-dimensional ultrasound which has been shown to be helpful in the clarification and delineation of both the normal and abnormal spine [**4,5**]. It is very important to differentiate the two subtypes of JLS because if a prenatal diagnosis of STD is made before viability, the prognosis and even the option of pregnancy termination should be offered to the parents [**4,19,20**]. Some authors suggest that post-natal high–resolution spiral CT-scan could complement the radiological examination for a better description of the thoracic and costal defects [**4,5**].

 Previous studies reported diagnosis of JLS in patients not known to be at risk for the condition in the first trimester - Hull et al. (2001) reported a diagnosed case of JLS, with a combination of three dimensional ultrasound and measurement of nuchal at 12 weeks of gestation and Kauffmann et al. (2003) described the first severe JLS case diagnosed during the first trimester of pregnancy in a family with no previous medical history of JLS [**21,5**]. However, in our case, the routine ultrasound at 6 and 12 weeks of gestation did not detect any anomalies. Perhaps the multiple large leiomyomas hindered the transvaginal ultrasound examination (**Fig. 2**).

 The case reported encloses in the first clinico-imagistic entity – the SCD phenotype of JLS. Although there was no previous history, the diagnose of SCD was established prenatal, at 18 weeks of gestation due to the three-dimensional ultrasound findings: thoracic and lumbar hemivertebrae with abnormal alignment of the vertebral bodies, absence of two right ribs and abnormal shaped ribs with kypho-scoliosis, but with biometric measurement appropriate for gestational age and no other malformations (**Fig. 1**). The patient underwent three-dimensional ultrasound at 24, 28 and 32 weeks of gestation, but it did not reveal different anomalies than the ones already described. 

 The patient gave birth at term by Cesarean section, to a male fetus, with a weight of 2700g, a length of 50cm and a calculated Apgar score of 9. The postpartum examination of the fetus confirmed the diagnose of SCD, established at 18 weeks of gestation. The evolution of the newborn was good - he had no respiratory difficulty, but he is still monitored for any signs of potential complications (i.e. – inguinal hernia which has an increased risk in males [**3,15**]). He will benefit from an experimental surgery involving expandable titanium ribs.

## Conclusions

Our case illustrates the importance of an accurate ultrasound examination, which can be hindered by multiple large leiomyomas, in order to diagnose and to differentiate the two subtypes of JLS. The SCD can have a favorable evolution with the appropriate perinatal and postpartum care. 
